# (Cardiac) complexity needs interaction

**DOI:** 10.1038/s41598-025-09349-5

**Published:** 2025-09-19

**Authors:** Tim De Coster

**Affiliations:** 1 Laboratory of Experimental Cardiology, Department of Cardiology, Leiden University Medical Center, Albinusdreef 2, PO 9600, Leiden, 2333 ZA The Netherlands; 2 Netherlands Heart Institute, Moreelsepark 1, Utrecht, 3511EP The Netherlands

**Keywords:** Cardiology, Computational biology and bioinformatics

## Abstract

The heart exemplifies a complex system where electrical, mechanical, and biochemical processes interact dynamically. Understanding cardiac complexity requires exploring these interactions across multiple levels, from cellular dynamics to organ-wide behavior. This collection examines key interactions in cardiology, including excitation-contraction coupling, arrhythmia quantification, electromechanical modeling, and imaging advances. By integrating mathematical, computational, and clinical perspectives, researchers uncover novel insights into heart function and dysfunction. Progress in this field relies on interdisciplinary collaboration, emphasizing the vital role of human interaction in translating discoveries into improved diagnostics and treatments.

## (Cardiac) complexity

**Fig. 1:**
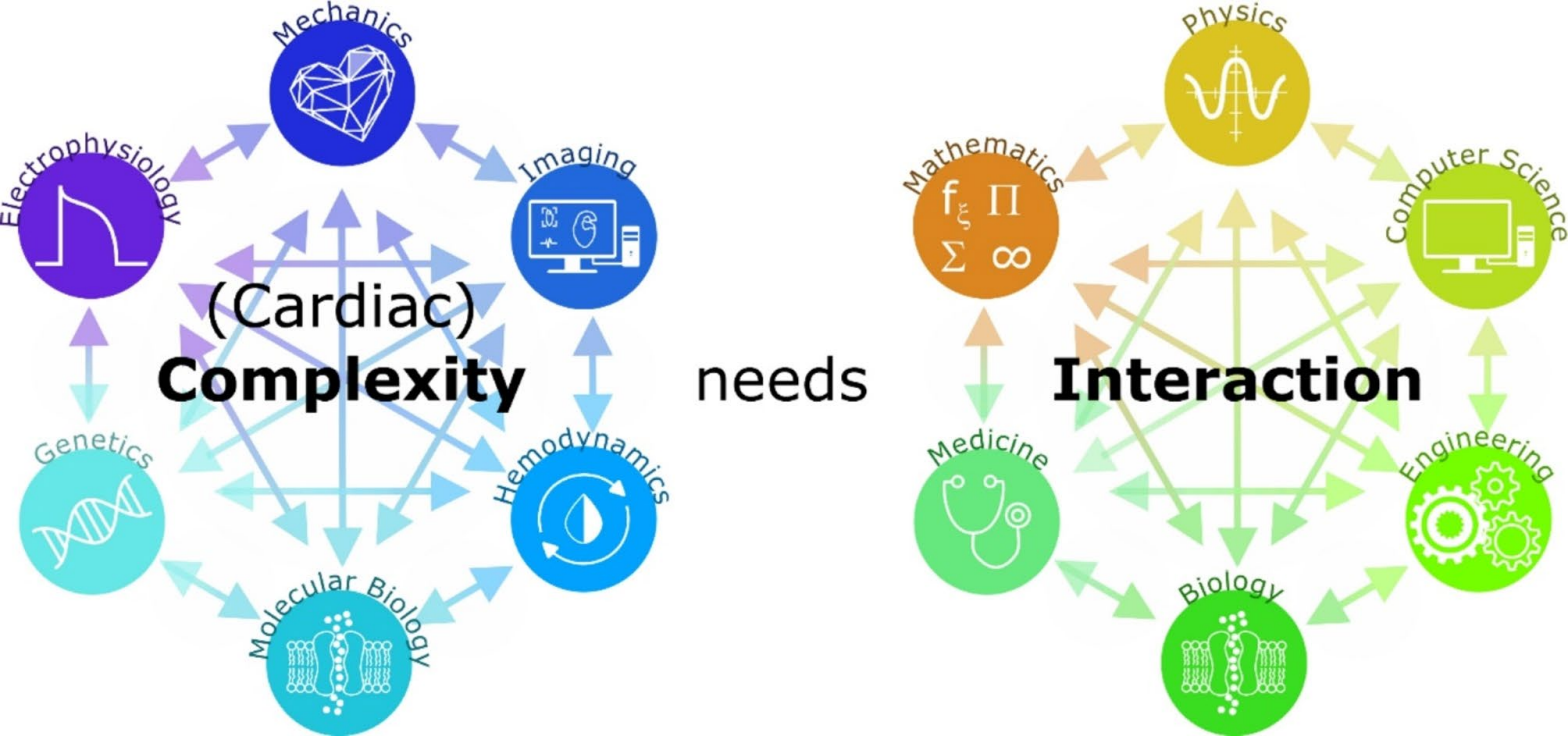
An overview of the complexity and interaction of different problems in cardiology, which can only be tackled using an equally complex interaction between scientific disciplines and expertise.

The Cambridge Dictionary defines *complexity* as “the state of having many parts and being difficult to understand or find an answer to.” Complexity arises in systems where numerous interconnected components interact in dynamic and often unpredictable ways. These systems can be found across disciplines from physics and biology to economics and social sciences. The heart is a prime example of such a system, where electrical, mechanical, and biochemical processes are intricately linked (Fig. 1). Importantly, complexity does not emerge from individual elements alone but from their interactions, which give rise to collective behaviors that cannot be easily inferred from the properties of the individual parts. Understanding these interactions is crucial in any field that deals with complex systems, as it provides deeper insight into their underlying principles and behaviors. In cardiology, gaining such insights helps us unravel the mechanisms behind heart function (e.g. contraction mechanisms) and dysfunction (e.g. heart rhythm disorders or arrhythmias), ultimately guiding the development of better treatments for patients.

Efforts to understand arrhythmias date back centuries, with early pioneers like Willem Einthoven, who discovered the mechanisms of the electrocardiogram^1^, laying the foundation for modern electrophysiology^2^. Theoretical frameworks, such as the Hodgkin-Huxley model of action potentials, provided key insights into cellular excitability^3^ while Panerai first described the link between electrical signals (Ca^2+^ dynamics) and mechanical contraction^4^. Observing these processes has evolved from invasive to advanced noninvasive imaging^5^. Nowadays, arrhythmia research increasingly relies on computational approaches, allowing for highly accurate simulations and visualizations of cardiac interactions. This advancement paves the way for tackling even more complex challenges in cardiology.

## Needs

To understand and unravel cardiac complexity and its many interactions, there are certain *needs* to be met. These needs can come in the deeper understanding of theory, the forming of new connections, development of new mapping/imaging techniques or systematic reviews of previous literature and patient cohorts. Such a wide variety of needs stems from the fact that the complex interactions in our heart occur across multiple levels, ranging from molecular and cellular dynamics to tissue organization and organ-wide electrical and mechanical behavior. A comprehensive understanding of these interconnected processes is essential not only for advancing fundamental cardiac research but also for translating insights into clinical applications. In this collection, multiple facets of the interactions in the heart are investigated:

A first interaction, the one between excitation patterns in cardiac tissue and their mathematical representation is explored by Arno et al.^6^ who introduce a novel framework for analyzing their complexity. Using “cardions”, quasiparticles that describe the dynamic behavior of wave breaks, conduction blocks, and vortices, Feynman-like diagrams can be constructed. These diagrams track the formation, annihilation, and recombination of these structures, providing a new way to visualize and quantify arrhythmic activity beyond the traditional phase singularity framework^7,8^.

Quantifying arrhythmias is also necessary for risk prediction, for example in the context of myocardial infarction and cell therapy. Using in silico models, Riebel et al.^9^ did exactly that and quantified how electrophysiological complexity—arising from factors like infarct stage, scar size, and Purkinje network integrity—affects spontaneous beats and re-entrant arrhythmias. Heterogeneity in injected cell populations and impaired Purkinje conduction were found to increase arrhythmic risk.

Besides excitation, there is also contraction in the heart. Zhou et al.^10^ study the interaction between electromechanical modeling and heart failure risk stratification, focusing on complexity in failing human cardiomyocytes. The researchers investigate in silico how changes in ionic currents and calcium handling contribute to proarrhythmic abnormalities. Their findings suggest that lower diastolic tension—rather than reduced contractility—is a key factor in arrhythmia susceptibility. This highlights the need for incorporating end-diastolic volume as a biomarker to improve current heart failure risk assessments^11^ acknowledging the intricate electromechanical interactions within the failing heart^12^.

Excitation-contraction leads to a measurable signal in the hospital used by doctors to assess the gravity of a patients’ condition. Lee et al.^13^ explored the interaction between signal analysis and risk prediction in atrial fibrillation (AF) by introducing the Complexity AF score. This score integrates AF burden, Electrical Burden, and Poincaré analysis to quantify the complexity of AF episodes. While traditional AF burden metrics focus on time spent in AF^14^ this study highlights the added value of electrical signal complexity in predicting AF progression and stability.

Interactions extend beyond cardiomyocytes alone and can also occur on a larger scale, such as between left atrial epicardial adipose tissue (EAT) and fibrosis. Skoda et al.^15^ investigated this interaction in AF patients, using advanced imaging techniques. Despite prior hypotheses suggesting a link between EAT infiltration and fibrotic remodeling^16^ the study found that the spatial overlap between EAT and fibrosis was minimal. Furthermore, this overlap was not significantly associated with the clinical stage of AF. These findings highlight the complexity of AF progression, indicating that while both EAT and fibrosis contribute to AF^17^ their direct anatomical interaction may not be a decisive factor in disease staging. This creates a possibility to tell something about the progression of disease where a major role might be reserved for electrophysical remodeling due to the interaction of EAT regions with healthy myocardium^18^.

Besides these five highlighted interactions, the possibilities stretch much further, encompassing for example interactions between advanced cardiovascular magnetic resonance imaging techniques and interventional electrophysiology^19^, between patient adherence and physician-prescription patterns in secondary stroke prevention^20^, or an assessment of the effectiveness and safety of catheter ablation in patients with structural heart disease and preserved left ventricular ejection fraction^21^.

## Interaction

The collective body of research describing complex *interactions* in the heart, presented in this collection, underscores the indispensable role of person-to-person *interactions*, multidisciplinary integration, and collaboration in advancing cardiac arrhythmia research (Fig. 1). The complexity inherent in cardiac function and dysfunction cannot be fully addressed by isolated disciplines. Instead, it requires the concerted efforts of clinicians, computational scientists, engineers, and researchers from various fields (just have a look at all the affiliations of the scientists that published in this collection). Continuous dialogue and collaboration are crucial for translating mathematical, physical, biological and computational insights into effective clinical interventions^22^.

In conclusion, while technological and computational advancements have significantly enriched our understanding of cardiac complexities, the essence of progress lies in human interaction. Interdisciplinary collaboration remains the cornerstone of developing comprehensive solutions to rhythm disorders. As we continue to integrate technology into cardiology, fostering robust communication and cooperation among diverse disciplines will be paramount in addressing the multifaceted challenges of cardiac arrhythmias.
